# Supplementary material for: Substantial undocumented infection facilitates the rapid dissemination of novel coronavirus (COVID-19)
Source: medRxiv. 2020 Feb 17:2020.02.14.20023127. Preprint. [Version 1] doi: 10.1101/2020.02.14.20023127 (PMC7239048; doi:10.1101/2020.02.14.20023127)
Supplement: Supplement 2020 [file 72157-2020.02.14.20023127-1.pdf]

Substantial undocumented infection facilitates the rapid dissemination of novel coronavirus (COVID-19)

Authors: Ruiyun Li[†,1], Sen Pei[†,2], Bin Chen[†,3], Yimeng Song[4], Tao Zhang[5], Wan Yang[6], Jeffrey Shaman[*,2]

Affiliations:

[1]MRC Centre for Global Infectious Disease Analysis, Department of Infectious Disease Epidemiology, School of Public Health, Faculty of Medicine, Imperial College London, London, W2 1PG, United Kingdom

[2]Department of Environmental Health Sciences, Mailman School of Public Health, Columbia University, New York, NY 10032, USA

[3]Department of Land, Air and Water Resources, University of California, Davis, CA 95616, USA

[4]Department of Urban Planning and Design, The University of Hong Kong, Hong Kong

[5]Ministry of Education Key Laboratory for Earth System Modeling, Department of Earth System Science, Tsinghua University, Beijing, 10084, P. R. China

[6]Department of Epidemiology, Mailman School of Public Health, Columbia University, New York, NY 10032, USA

Supplementary Appendix

1. Model configuration

The initial prior ranges of the parameters for the model (Equations 1-5 in the main text) are drawn from uniform distributions with the following prior ranges:

- β: the transmission rate of symptomatically infected patients. $0.6 \leq \beta \leq 1.5$
- μ: the multiplicative factor reducing the transmission rate of unreported infected patients. $0.2 \leq \mu \leq 1$.
- θ: the multiplicative factor to adjust mobility data estimates of human movement between cities. $1 \leq \theta \leq 1.75$.
- Z: the mean latency period. $2\ days \leq Z \leq 5\ days$.
- α: the fraction of infections that develop severe symptoms. $0.02 \leq \alpha \leq 0.8$.
- D: the average duration of infection for infected patients. $2\ days \leq D \leq 5\ days$.

We model the transmission of COVID-19 in China **from January 10th, 2020 to January 23rd, 2020.** Strict travel restrictions were implemented in several Chinese cities beginning January 23rd, 2020. As a result, the 2018 mobility data we use are likely not representative of inter-city human movement after January 23rd.

2. Model-inference framework

We infer model epidemiological parameters using an iterated filtering (IF) approach (1-3). To account for the high-dimensionality of the model, rather than employ a particle filter (4) (i.e. a sequential Monte Carlo approach), we used an efficient data assimilation method – the Ensemble Adjustment Kalman Filter (EAKF) (5) within the IF algorithm. Particle filters require a large number of particles (6); however, the EAKF can generate similar results using only hundreds of ensemble members (7). For this IF-EAKF approach, an ensemble of system states, which represent the distribution of parameters, is repeatedly adjusted using the EAKF in a series of iterations, during which the variance of the parameters is gradually tuned down. Through this process, the distribution of parameters is iteratively optimized per observations and converges to values that approach maximum likelihood. Details of the IF-EAKF algorithm can be found in Ref. (3).

We used the daily number of reported cases in city i on a given day t, y_i^t, as observations. For each y_i^t, we assume a heuristic observation error variance (OEV):

$$y_i^t = \max\left(4, \frac{(y_i^t)^2}{5}\right).$$

Similar forms of OEV have been successfully used for inference and forecasting for a range of infectious diseases including influenza (8-12), Ebola (13), West Nile virus (14) and respiratory syncytial virus (15). In total, 10 rounds of EAKF

were performed for the inference. After each round of assimilation with the EAKF, the standard deviation for each parameter was reduced by 10%.

3. Calculation of R_e in Wuhan city

We calculated the effective reproductive number R_e in Wuhan city using the inferred parameters. Specifically, R_e is the largest eigenvalue of the next-generation matrix (NGM) (16,17). Define $X = [E, I^r, I^u]^T$ and $Y = [S, R]^T$. The vectors for new infection and other rates are:

$$\mathcal{F} = \begin{bmatrix} \frac{\beta S I^r}{N} + \frac{\mu \beta S I^u}{N} \\ 0 \\ 0 \end{bmatrix}, \mathcal{V} = \begin{bmatrix} \frac{E}{Z} \\ \frac{I^r}{D} - \frac{\alpha E}{Z} \\ \frac{I^u}{D} - \frac{(1-\alpha)E}{Z} \end{bmatrix}.$$

The disease-free equilibrium is $x_0 = [0,0,0,N,0]^T$. We then have

$$F = \frac{\partial \mathcal{F}}{dX}\bigg|_{x_0} = \begin{pmatrix} 0 & \beta & \mu\beta \\ 0 & 0 & 0 \\ 0 & 0 & 0 \end{pmatrix}$$

and

$$V = \frac{\partial \mathcal{V}}{dX}\bigg|_{x_0} = \begin{pmatrix} \frac{1}{Z} & 0 & 0 \\ -\frac{\alpha}{Z} & \frac{1}{D} & 0 \\ -\frac{1-\alpha}{Z} & 0 & \frac{1}{D} \end{pmatrix}.$$

The NGM is $K = FV^{-1}$. R_e is then computed as the leading eigenvalue of the NGM K, i.e.,

$$R_e = \alpha\beta D + (1-\alpha)\mu\beta D.$$

4. Synthetic test

Before applying the model-inference framework to the observed COVID-19 incidence data, we tested the model-inference framework using model-generated outbreaks. Specifically, we generated a synthetic outbreak using a free simulation of the metapopulation model with a set of specified parameters. We then ran IF-EAKF inference using the daily cases for each city, as generated in stochastic free simulation, as observations. The aim is to determine whether model inference framework can ingest observations and recover the specified parameters. This assessment of the performance of the inference algorithm also allows inspection of the sensitivities of the inference results to model assumptions.

4.1. Accuracy of parameter estimation

We first generated a synthetic outbreak using the following parameter values: $\beta = 1.0$, $\mu = 0.8$, $\theta = 1.5$, $Z = 4$ days, $\alpha = 0.1$, $D = 4$ days, $T_d = 4.6$ days. For the IF, a 300-member ensemble was used. Priors of variables and parameters were drawn from the ranges reported in Section 1 of this Supplementary Appendix using a Latin Hypercubic Sampling algorithm. We used $Seed_{max} = 500$ in Wuhan city to initiate the outbreak. During inference, the seeding parameter $Seed_{max}$ was also set as 500.

To account for stochastic effects, we applied the IF-EAKF inference algorithm 300 times and report the distributions of estimated parameters. All parameters were accurately estimated (Fig. S1) and the effective reproductive number R_e was recovered (Fig. S2).

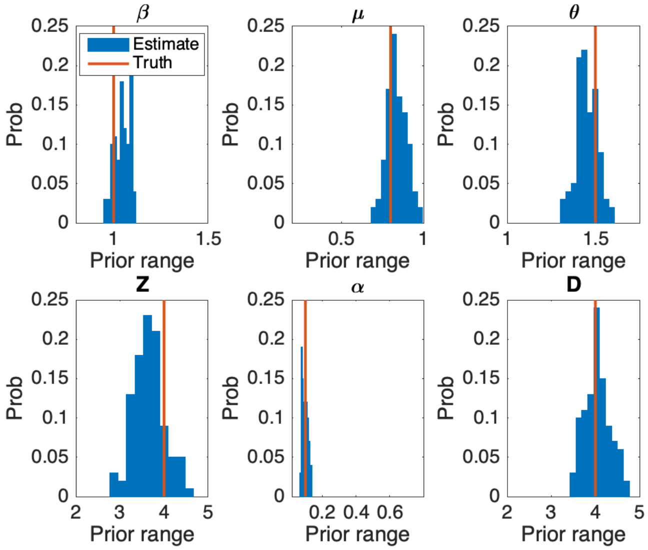

Fig. S1. Accuracy of parameter estimation. The actual parameters used in generating the synthetic outbreak are depicted by vertical red lines. Blue bars represent the distribution of the posterior parameter estimates. The ranges of the x-axis are set as the initial prior parameter ranges.

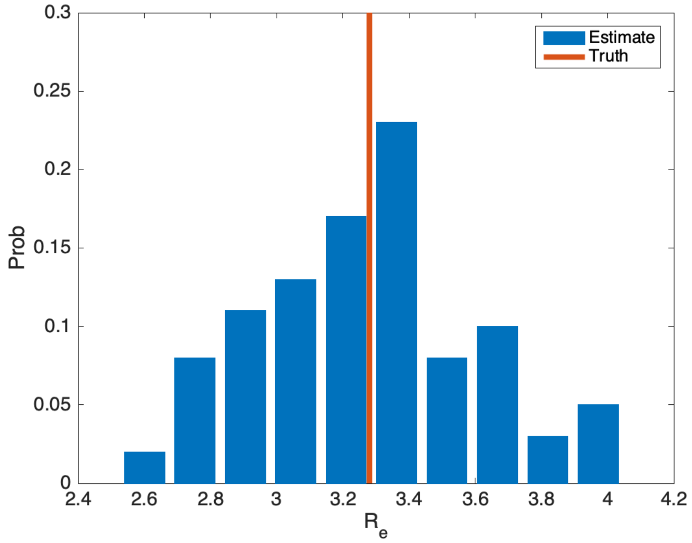

Fig. S2. Comparison of the actual R_e (vertical red line) and the distribution of estimated R_e (blue bars).

To further validate the inference approach, we also tested the system on another synthetic outbreak generated with a lower R_e ($\beta = 1.0$, $\mu = 0.6$, $\theta = 1.5$, $Z = 4$ days, $\alpha = 0.2$, $D = 4$ days, $T_d = 4.6$ days). Again, epidemiological parameters and R_e were captured by the estimated distributions (see Figs. S3-4).

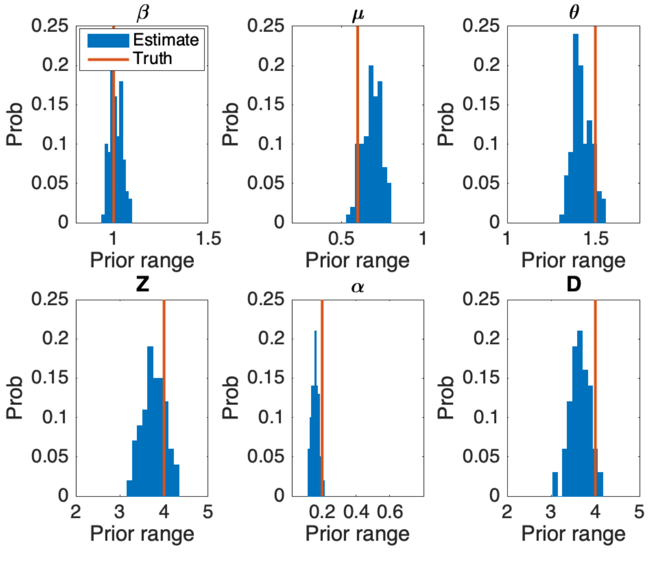

Fig. S3. Accuracy of parameter estimation. The actual parameters used in generating the synthetic outbreak are depicted by vertical red lines. Blue bars represent the distribution of the posterior parameter estimates. The ranges of the x-axis are set as the initial prior parameter ranges.

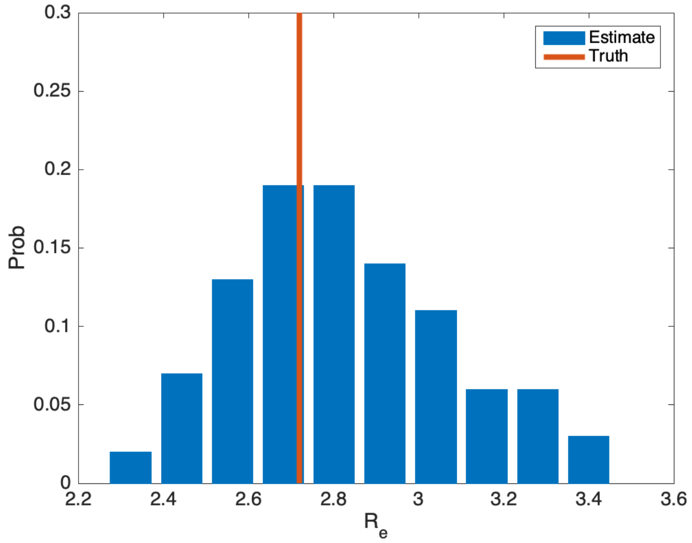

Fig. S4. Comparison of the actual R_e (vertical red line) and the distribution of estimated R_e (blue bars).

A third synthetic outbreak with a higher reporting rate ($\alpha = 0.5$) was additionally tested ($\beta = 1.0$, $\mu = 0.6$, $\theta = 1.5$, $Z = 4$ days, $\alpha = 0.5$, $D = 4$ days, $T_d = 4.6$ days). As shown in Figs. S5-6, this high reporting rate was also accurately estimated.

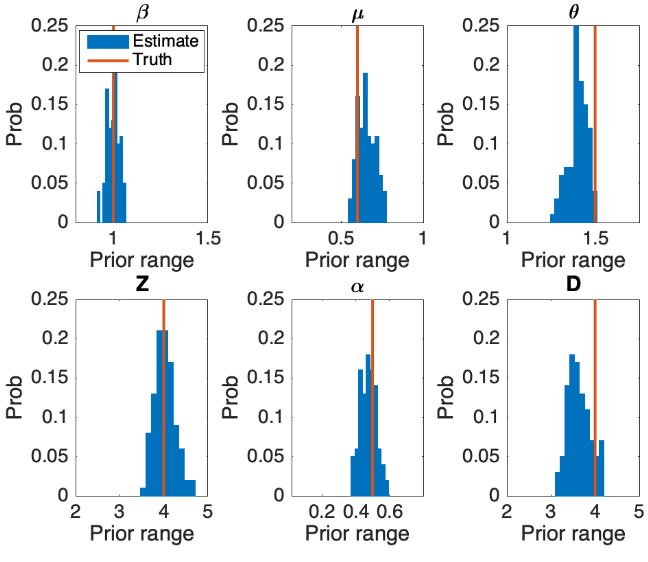

Fig. S5. Accuracy of parameter estimation. The actual parameters used in generating the synthetic outbreak are depicted by vertical red lines. Blue bars represent the distribution of the posterior parameter estimates. The ranges of the x-axis are set as the initial prior parameter ranges.

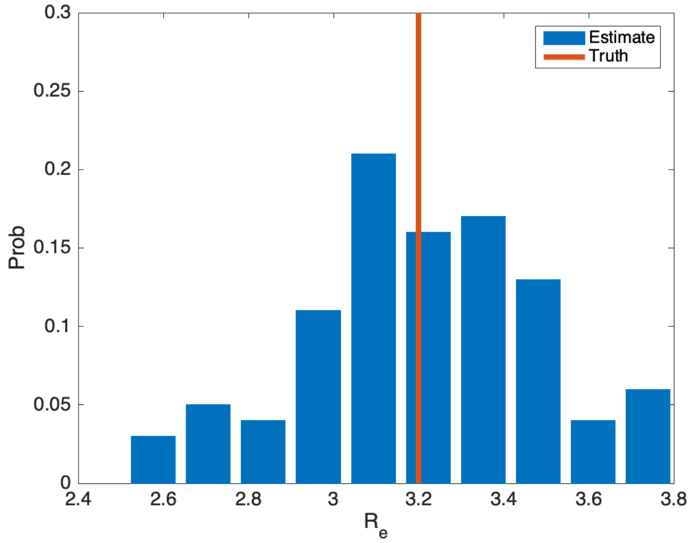

Fig. S6. Comparison of the actual R_e (vertical red line) and the distribution of estimated R_e (blue bars).

4.2. Sensitivity of parameter estimation to seeding

As the numbers of exposed (E) and unreported infected (I^u) population are unobserved, we estimated these state variables along with the other parameters/variables using the IF-EAKF approach. In particular, E and I^u may be sensitive to the initial seeding ($Seed_{max}$). It is thus critical to examine the sensitivity of the overall parameter estimation to the seeding parameter $Seed_{max}$. We repeated the inference shown in Figs. S1-2 using a higher seeding parameter $Seed_{max} = 1{,}000$ (the true $Seed_{max}$ is 500). As shown in Figs. S7-8, with a mis-specified, higher prior for $Seed_{max}$, the reporting rate α is slightly underestimated. However, the estimation biases for other parameters are limited and R_0 is also identified.

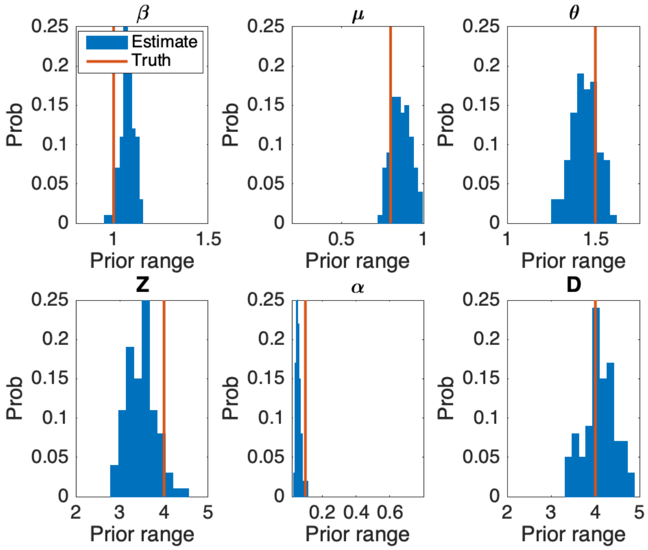

Fig. S7. Accuracy of parameter estimation. The actual parameters used in generating the synthetic outbreak are depicted by vertical red lines. Blue bars represent the distribution of the posterior parameter estimates. The ranges of the x-axis are set as the initial prior parameter ranges. The actual seeding parameter for the synthetic outbreak is $Seed_{max} = 500$, while $Seed_{max}$ was set as 1,000 during inference.

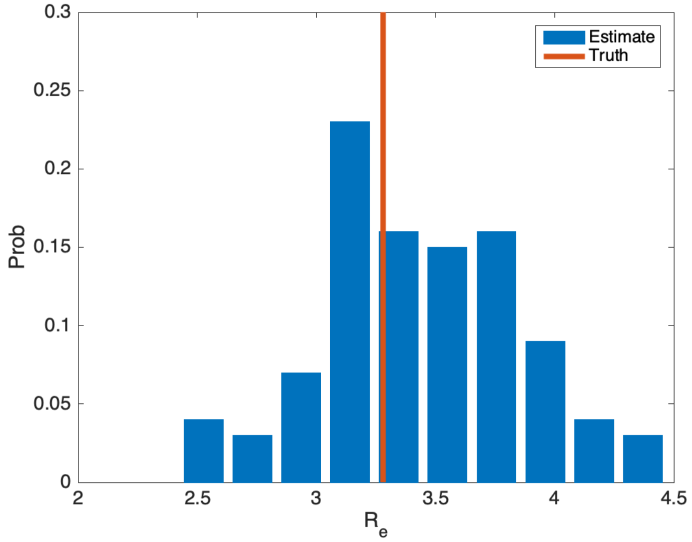

Fig. S8. Comparison of the actual R_e (vertical red line) and the distribution of estimated R_e (blue bars).

5. Inference using documented cases

We used the reported cases from 375 Chinese cities during January 10th, 2020 to January 23rd, 2020 to infer model parameters. In total, 770 cases were reported, with 454 cases in Wuhan city and 500 in Hubei province.

We tested a range of seeding parameters ($Seed_{max} =$ 3000, 4000, 5000 and 6000) and reporting delays ($T_d =$ 6, 7, 8, 9, 10, 11 and 12 days). For each combination of seeding and reporting delay parameters, we ran the inference 300 times. To validate the estimates, we generated 300 outbreaks using the inferred parameters and corresponding seeding parameters, and then compared the distributions of simulated new cases in all cities with reported case observations. The goodness-of-fit was measured using log-likelihood (LL). The log-likelihood is computed using a Poisson distribution fitted to the simulation outcomes in each city. For each observation, we calculate the logarithmic value of the weight assigned to a +/-2 interval around the reported incidence. LL is the sum of these values. Inference results for the best-fitting model with the maximum LL ($Seed_{max} = 5000$, $T_d = 10$ days, LL=-176.38) are shown in Table 1 of the main text, and model fitting for this inference solution is shown in Fig. 1 of the main text.

6. Spatial movement of COVID-19 in China

Using the best-fitting model ($Seed_{max} = 5000$, $T_d = 10$ days), we generated 300 simulated outbreaks starting January 10th until January 23rd. We computed the daily number of cities with cumulative incidence ≥ 10, and compared these distributions with the reported numbers of invaded cities during the same period (Fig. S9). The observations and simulations are in good agreement.

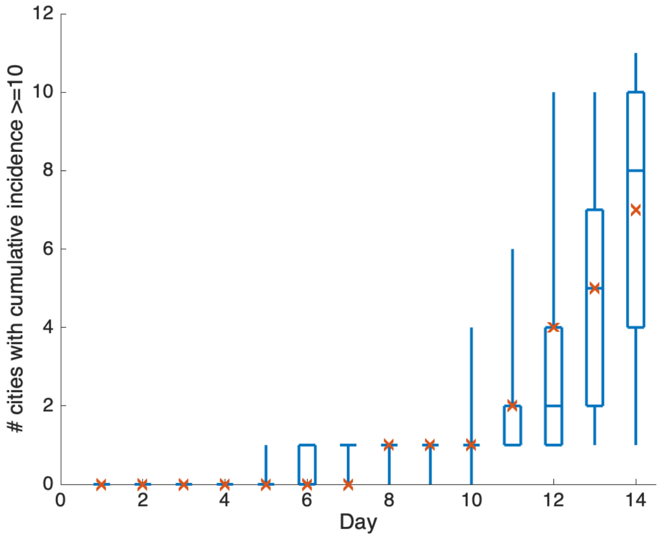

Fig. S9. Model-generated distributions of the number of cities with cumulative incidence ≥ 10 at each day from January 10th to January 23rd. Red crosses are reported numbers until January 23rd.

7. Sensitivity of parameter estimates

Distributions for estimated parameters and R_0 for different settings of $Seed_{max}$ and T_d are shown in Fig. S10. Estimations of β, μ, θ, Z and D are robust to different settings of $Seed_{max}$ and T_d. Estimation of the reporting rate α is more sensitive to $Seed_{max}$ and T_d, but generally falls within the range between 0.1 and 0.2. The effective reproductive number R_e is also robustly estimated between 2.0 and 2.5.

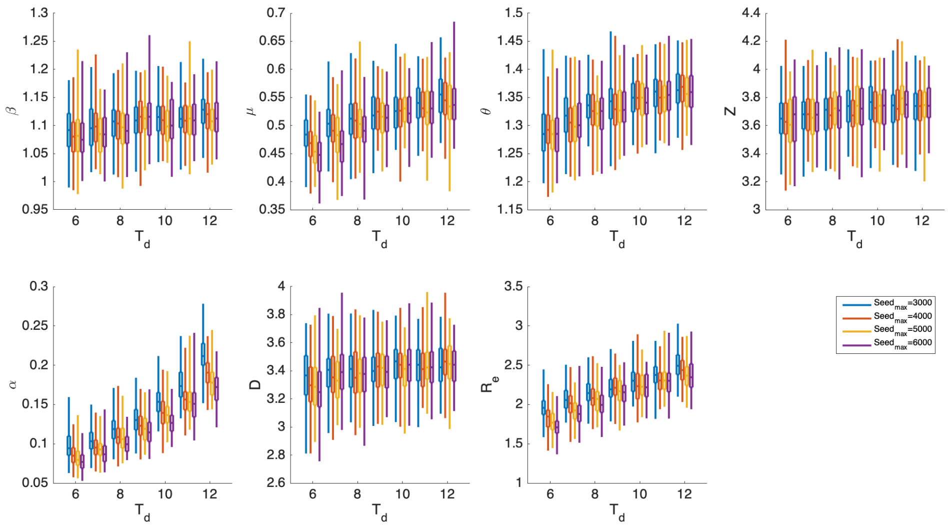

Fig. S10. Distributions of estimated parameters and R_0 for different settings of $Seed_{max}$ and T_d. Boxes show median and interquartile values and whiskers indicate the 95% CIs.

8. Inference of model parameters after January 23rd

We modeled the transmission of COVID-19 in China after implementation of control measures on January 23. These control measures included: travel restrictions imposed between major cities and Wuhan; self-quarantine and contact precautions advocated by the government; and more available rapid testing for infection confirmation (18-19). These control measures along with changes in care-seeking behavior due to increased awareness of the virus and increased personal protection behavior (e.g. wearing of facemasks, self-isolation when sick), likely altered the epidemiological characteristics of the outbreak after January 23. To quantify these differences, we re-estimated the system parameters using the metapopulation model-inference framework and city-level daily cases reported between January 24 and February 8. As inter-city mobility was restricted, we set $\theta = 0$. In addition, to represent reduced person-to-person contact and increased infection detection, we updated the initial priors for β and α to $[0.2, 1.0]$ and $[0.2, 1.0]$, respectively.

We tested a range of reporting delays, T_d, from 3 days to 10 days, given the fact that this delay may have been reduced due to the increasing availability of assays and changes in medical care-seeking behavior. We used the daily reported cases in all cities to compute the log-likelihood.

In order to reflect the rapid change in control efforts, we inferred model parameters during two overlapping periods: January 24 to February 3 and January 24 to February 8. For these periods, the best-fitting models are shown in Fig. S11 and Fig. S12. Estimated parameters, R_e and goodness-of-fit are reported in Table 2 of the main text.

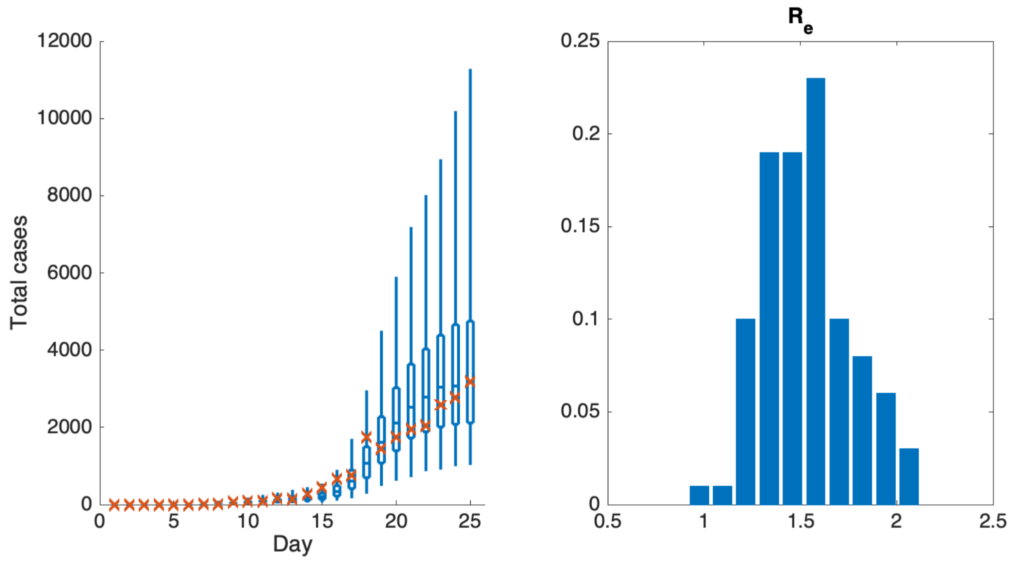

Fig. S11. Model fitting ($T_d = 5$ days) to documented cases in all cities through February 3, 2020 (left). The distribution of estimated R_e is shown in the right panel.

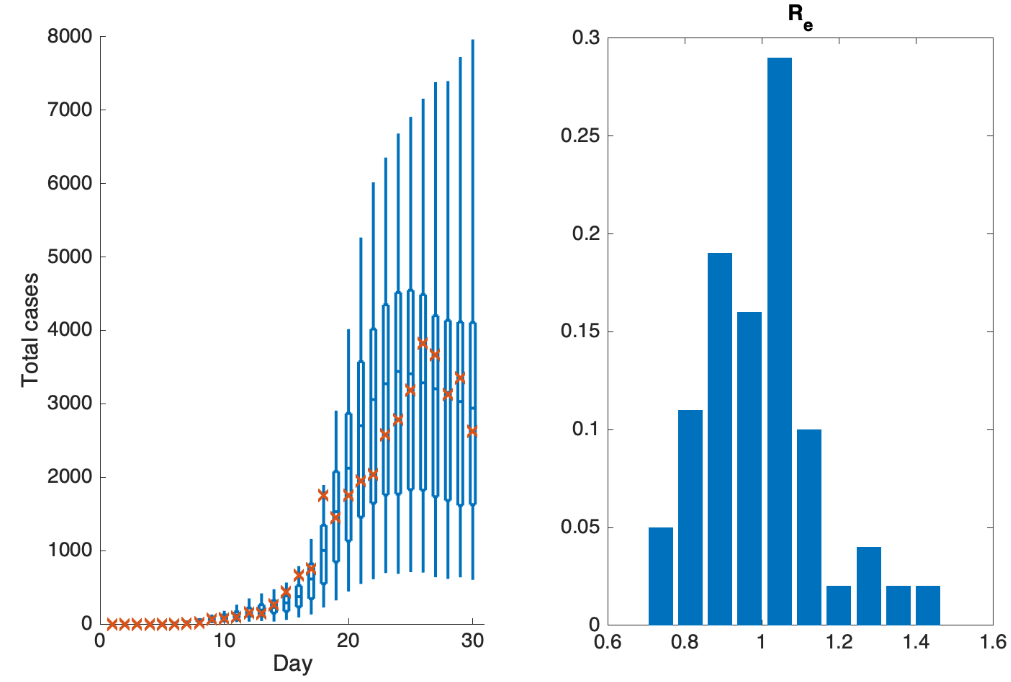

Fig. S12. Model fitting ($T_d = 5$ days) to documented cases in all cities through February 8, 2020 (left). The distribution of estimated R_e is shown in the right panel.

To further test the sensitivity of this parameter estimation during January 24 and February 8 to the initial priors, we repeated the inference using a different set of priors for β and α: $\beta \in [0.2, 1.2]$ and $\alpha \in [0.2, 0.8]$. These estimated parameters are shown in Table S1. The estimated parameters generally match with the estimates in Table 2 of the main text.

Table S1. Best-fit model posterior estimates of key epidemiological parameters for simulation of the model without travel between cities from January 23, 2020 to February 8, 2020 and using a different set of priors: $\beta \in [0.2, 1.2]$ and $\alpha \in [0.2, 0.8]$.

Parameter	Meaning	Median estimates (95% CIs)
β	Transmission rate	0.36 (0.29, 0.57)
μ	Relative transmission rate	0.41 (0.28, 0.70)
Z	Latency period	3.47 (3.19, 3.87)
α	Reporting rate	0.68 (0.46, 0.75)
D	Infectious period	3.50 (3.18, 3.76)
R_e	Effective reproductive number	1.03 (0.81, 1.53)

9. Independent model validation using infection rates among evacuees to other countries

A recent study (20) summarized infection rates in evacuees to Singapore, South Korea, Japan and Germany at the end of January. The average infection rate was reported as 1.39%. Based on this estimate, we performed two independent tests to corroborate the parameters inferred by the metapopulation model.

1). According to the official report, around 5 million people left Wuhan city before January 23. The total population in Wuhan city after January 23 (when travel restrictions were imposed) is therefore around 6 million. A 1.39% infection rate suggests an estimated 83,400 infections prior to February 1 in Wuhan. Simulation with the metapopulation model using inferred parameters produce a total infected population of 106,010 (95% CI [22,005, 201,535]) before Feb 1, which generally matches the estimated 83,400 infections in magnitude.

2). In our model, infections occurring prior to February 1 will be documented with a reporting delay (as inferred, an average of 10 days before January 23 and 5 days after January 23). For simplicity, we assume the reporting delay for each person is constant. Based on this assumption, infections before February 1 should continue appearing until February 6. The cumulative confirmed cases for February 6 in Wuhan is 11,577, which suggests a **reported** infection rate of 11,577/6 millions = 0.19%. Compared with the infection rate 1.39% (20), the reporting rate in Wuhan should be 0.19%/1.39%=13.67%. This estimate agrees with our inferred reporting rate of 14% before January 23.

References

1. Ionides EL, Breto C, King AA. Inference for nonlinear dynamical systems. Proc. Natl. Acad. Sci. U. S. A. 2006;103:18438–18443.
2. King AA, Ionides EL, Pascual M, et al. Inapparent infections and cholera dynamics. Nature. 2008;454: 877–880.
3. Pei S, Morone F, Liljeros F, et al. Inference and control of the nosocomial transmission of methicillin-resistant Staphylococcus aureus. eLife. 2018;7:e40977.
4. Arulampalam MS, Maskell S, Gordon N, et al. A tutorial on particle filters for online nonlinear/non-Gaussian Bayesian tracking. IEEE Trans. Signal. Process**.** 2002;50: 74–188.
5. Anderson JL. An Ensemble Adjustment Kalman Filter for Data Assimilation. Mon. Weather Rev*.* 2001;129: 2884–2903.
6. Snyder C, Bengtsson T, Bickel P et al. Obstacles to high-dimensional particle filtering. Mon. Weather Rev. 2008;136: 4629–4640.
7. Yang W, Karspeck A, Shaman J. Comparison of filtering methods for the modeling and retrospective forecasting of influenza epidemics. PLoS Comput. Biol. 2014;10: e1003583.
8. Pei S, Kandula S, Yang W et al. Forecasting the spatial transmission of influenza in the United States. Proc. Natl. Acad. Sci. U. S. A. 2018;115:2752–2757.
9. Shaman J, Karspeck A. Forecasting seasonal outbreaks of influenza. Proc. Natl. Acad. Sci. U. S. A. 2012;109: 20425–30.
10. Shaman J, Karspeck A, Yang W. *et al.* Real-time influenza forecasts during the 2012–2013 season. Nat. Commun. 2013;4: 1057–1062.
11. Yang W, Lipsitch M, Shaman J. Inference of seasonal and pandemic influenza transmission dynamics. Proc. Natl. Acad. Sci. U. S. A. 2015;112: 201415012.
12. Pei S, Shaman J. Counteracting structural errors in ensemble forecast of influenza outbreaks. Nat. Commun. 2017;8: 925.
13. Shaman J, Yang W, Kandula S. Inference and Forecast of the Current West African Ebola Outbreak in Guinea, Sierra Leone and Liberia. PLoS Curr. 2014;6: ecurrents.outbreaks.3408774290b1a0f2dd7cae877c8b8f.
14. DeFelice NB, Little E, Campbell SR, et al.. Ensemble forecast of human West Nile virus cases and mosquito infection rates. Nat. Commun. 2017;8: 14592.
15. Reis J, Shaman J. Retrospective parameter estimation and forecast of respiratory syncytial virus in the United States. PLoS Comput. Biol. 2016;12: e1005133.
16. Diekmann O, Heesterbeek JAP, Metz JA. On the definition and the computation of the basic reproduction ratio R0 in models for infectious diseases in heterogeneous populations. J. Math. Biol. 1990;28: 365–382.
17. Diekmann O, Heesterbeek JAP, Roberts MG. The construction of next-generation matrices for compartmental epidemic models. J. Roy. Soc. Interface. 2010;7: 873–885.

18. The 8th Press Conference on the Prevention and Control of COVID-19. Health Commission of Hubei Province. Available: http://wjw.hubei.gov.cn/fbjd/dtyw/202001/t20200130_2016544.shtml
19. The 9th Press Conference on the Prevention and Control of COVID-19. Health Commission of Hubei Province. Available: http://wjw.hubei.gov.cn/fbjd/dtyw/202001/t20200131_2017018.shtml
20. Lai, S., Bogoch, I., Ruktanonchai, N., Watts, A., Li, Y., Yu, J., Lv, X., Yang, W., Yu, H., Khan, K. and Li, Z., 2020. Assessing spread risk of Wuhan novel coronavirus within and beyond China, January-April 2020: a travel network-based modelling study. medRxiv. doi: https://doi.org/10.1101/2020.02.04.20020479
